# Spinyhead Croaker Germ Cells Gene *dnd* Visualizes Primordial Germ Cells in Medaka

**DOI:** 10.3390/life12081226

**Published:** 2022-08-12

**Authors:** Cong Xu, Yu Li, Zhengshun Wen, Muhammad Jawad, Lang Gui, Mingyou Li

**Affiliations:** 1 Key Laboratory of Integrated Rice-Fish Farming, Ministry of Agriculture and Rural Affairs, Shanghai Ocean University, Shanghai 201306, China; 2 Key Laboratory of Exploration and Utilization of Aquatic Genetic Resources, Ministry of Education, Shanghai Ocean University, Shanghai 201306, China; 3 Main Building, QiLu Innovalley Incubator, High-Tech Industry Development Zone, Jinan 250101, China; 4 Zhejiang Provincial Engineering Technology Research Center of Marine Biomedical Products, School of Food Science and Pharmaceutics, Zhejiang Ocean University, Zhoushan 316022, China

**Keywords:** *dnd*, *Collichthys lucidus*, PGC

## Abstract

**Highlights:**

- *Cldnd* was specifically expressed in adult gonads.
- *Cldnd* RNA was enriched in spermatogonia and early oocytes and decreased with gametogenesis.
- *Gfp-Cldnd* 3′ UTR mRNA could identify primordial germ cells in medaka.

**Abstract:**

Spinyhead croaker (*Collichthys lucidus*) is an economically important fish suffering from population decline caused by overfishing and habitat destruction. Researches on the development of primordial germ cell (PGC) and reproduction biology were an emergency for the long-term conservation of the involved species. *Dead end* (*dnd*) gene plays an indispensable role in PGC specification, maintenance, and development. In the current study, we report the cloning and expression patterns of *dnd* in *C. lucidus* (*Cldnd*). RT-PCR analysis revealed that *Cldnd* was specifically expressed in both sexual gonads. In the ovary, *Cldnd* RNA was uniformly distributed in the oocytes and abundant in oogonia, and gradually decreased with oogenesis. A similar expression pattern was also detected in testis. Dual fluorescent in situ hybridization of *Cldnd* and *Clvasa* demonstrated that they almost had the same distribution except in oocytes at stage I, in which the *vasa* RNA aggregated into some particles. Furthermore, *Cldnd* 3′ UTR was sufficient to guide the Green Fluorescent Protein (GFP) specifically and stably expressed in the PGCs of medaka. These findings offer insight into that *Cldnd* is an evolutionarily conserved germline-specific gene and even a potential candidate for PGC manipulation in *C. lucidus*.

## 1. Introduction

Primordial germ cells (PGCs) are the germline precursor which are specified from somatic cells and migrate into the genital ridges, ensuring population and intergenerational inheritance of genetic information. The specification, migration, proliferation, and differentiation of PGCs are precisely regulated by a sophisticated molecular network. As well, it has been demonstrated that a number of functional genes play critical roles in the development of PGCs [1,2]. Among these, *dead end* (*dnd*) gene attaches attention not only for its indispensable role in PGC development, but also for its successful application to prepare germ cell-deficient recipients for germ cell transplantation [3].

Dnd was initially identified in zebrafish as an RNA-binding protein that is specifically expressed in germ cells causing aberrant PGC migration and germ cell-deficient when blocked by specific antisense morpholinos [4]. Additionally, homologous *dnd* genes are characterized in diverse vertebrates, such as mice (*Mus musculus*) [5], frog (*Xenopus laevis*) [6], chickens (*Gallus gallus*) [7], medaka (*Oryzias latipes*) [8], Chinese sturgeon (*Acipenser sinensis*) [9], gibel carp (*Carassius Gibelio*) [10], orange-spotted grouper (*Epinephelus coioides*) [11] and Celebes medaka (*Oryzias* *celebensis*) [12]. All these studies suggest that *dnd* is primarily expressed in PGCs during embryogenesis, but it is shown sexually dimorphic in adult gonads across species. In mice, *dnd* is only detectable in testis [5], whereas it is restricted to the ovary in frog [6]. *Dnd* occurs in germ cells of both sexes in medaka [8], zebrafish (*Danio rerio*) [4], carp (*Carassius gibelio*) [10], turbot (*Scophthalmus maximus*) [13] and starry flounder (*Platichthys stellatus*) [14].

It is proven that *dnd* is essential for primordial germ cell (PGC) specification, maintenance, and development. In mice, *dnd* mutation causes germ cell loss or testicular germ cell tumors [5]. *Dnd* knockdown leads to abnormal migration and loss of PGCs in frogs [6] and zebrafish [4,15,16]. Especially in medaka, *dnd* is a specifier that can abolish or increase the PGC in a dosage-dependent manner [1]. *Dnd* is of great importance in the capability of the PGCs fate protection from somatic differentiation [16,17]. *Dnd* has been broadly used to produce PGC-deficiency individuals for the application of surrogate broodstock technology (SBT) in some teleosts, such as medaka [18], Atlantic cod (*Gadus morhua*) [19], olive flounder (*Paralichthys olivaceus*) [20], carp [21,22], grouper [11], salmon (*Salmo salar*) [23] and rainbow trout (*Oncorhynchus mykiss*) [24]. The SBT [18,25,26,27] is recognized as a promising approach for preventing endangered species or expanding valuable fish species from suffering population decline. Furthermore, the fact that rescue of *dnd* crispant embryos enables inherited sterile offspring production benefits the actual application of sterile fish as an approach to avoid genetic introgression in the aquaculture industry [28]. 

Spinyhead croaker (*Collichthys lucidus*) is an economically important fish in the east coastal areas and estuaries of China. Due to its delicious taste and high nutritional value, it is popular among other fish species [29]. The market-oriented demand that ascends gradually pushes up the price of *C. lucidus* and leads to its population decline due to overfishing [30]. Therefore, large-scale artificial reproduction is imperative to compensate for the limited natural reproductive rate. Nevertheless, research on *C. lucidus* is mainly limited to morphology, immune-related gene identification, population genetics, genome assembly, and transcriptome assembly with few reports on sex determination [29,31,32,33,34,35,36]. In the previous study, *C. lucidus* germ cells marker gene *vasa* was isolated by us [37]. In the present study, another gem cells gene *dnd(Cldnd)* was cloned, and ClDnd protein was highly conserved across species. *Cldnd* was only expressed in the germ cells of both sexual. Furthermore, *Cldnd* 3′ UTR was sufficient to guide the Green Fluorescent Protein (GFP) specifically and stably expressed in the PGCs in medaka, suggesting that *Cldnd* is an evolutionarily conserved germline-specific gene and even a potential candidate for PGC manipulation in *C. lucidus*.

## 2. Materials and Methods

### 2.1. Fish and Sampling

*C. lucidus* were sampled from the East China Sea in Ningde, Fujian province, China. For the total RNA extraction, tissue samples from the kidney, gill, intestines, brain, heart, ovary and testis were immediately collected and flash-frozen in liquid nitrogen. Gonad tissues were fixed for the frozen section. The *O. latipes* was kept at 26 °C in a 14-h light/10-h dark cycle and its embryos were maintained and staged as described [38]. All experiments were conducted in strict accordance with the guidance of the Committee for Laboratory Animal Research at Shanghai Ocean University.

### 2.2. RNA Isolation and cDNA Synthesis

Total RNA was isolated from the adult tissues mentioned above by using TRIzol^®^ Reagent (Invitrogen, Carlsbad, CA, USA), and the integrity of the RNA was detected by 1% agarose gel electrophoresis with ethidium bromide (EB) staining. The first-strand cDNA was synthesized with 1 μg total RNA and oligo dT_18_ primers following the manufacturer’s manual for the M-MLV reverse transcription kit (Takara, Shiga, Japan). Additionally, using BD SMART^®^ RACEs kit (Clontech, Beijing, China), two cDNA libraries from the ovary and testis were constructed for the full-length sequence amplification.

### 2.3. Molecular Cloning and Analysis of C. lucidus dnd Gene

**Figure 1:**
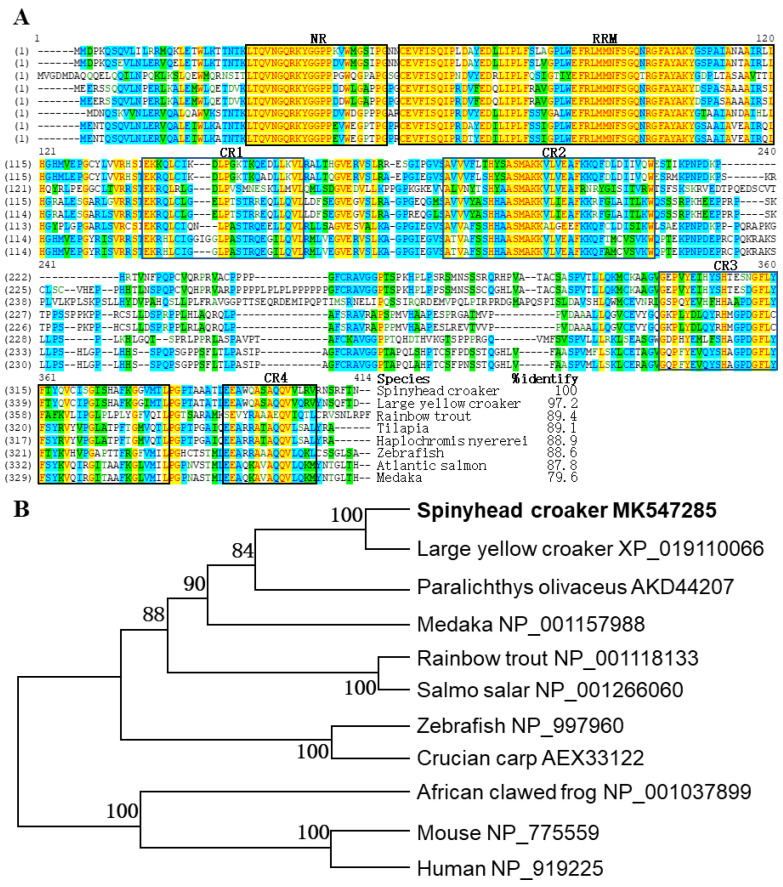
Multiple alignment and phylogenetic tree of Dnd proteins. (**A**) Multiple alignment of Dnd proteins. Species and overall sequence identity values compared to the ClDnd protein were at the end of the alignment. RNA recognition motif (RRM) and five conserved regions were indicated in the frame (black). (**B**) Phylogenetic tree of Dnd proteins. Bootstrap values were given. Accession numbers followed organism.

To identify the full-length sequence of *Cldnd,* the classic RACEs approach was performed. Briefly, according to the conserved amino acid sequences (WEFRLMM and MAKKVLVE) of Dnd protein, a pair of degenerate primers were designed to explore the corresponding fragment of *Cldnd* (Figure 1A). Subsequently, specific RACEs primers were designed based on the above fragment to amplify the 5′ and 3′ cDNA ends of *Cldnd* by using BD SMART^®^ RACEs kit (Clontech). All the primers used in the present research were listed in Table 1. Finally, the full-length mRNA sequence of *Cldnd* was assembled via DNAMAN and aligned to the Dnd protein across the examined species by Vector NTI Suite 11.0 (Invitrogen). Dnd proteins in vertebrates, from teleosts, amphibians to mammals, were achieved from NCBI (https://www.ncbi.nlm.nih.gov/protein) to investigate ClDnd conservatism in evolution. Afterwards, the phylogenetic tree was constructed by the neighbor-joining method based on Mega 7.0 package.

### 2.4. RT-PCR

To confirm the tissue distribution of *Cldnd*, a pair of gene-specific primers targeting to its coding sequence (CDS) together with *β-actin* as an internal control was designed and RT-PCR was carried out by using Ex-Taq^®^ (Takara, Beijing, China). PCR program was performed in a 25 μL reaction system for 28 cycles: denaturation at 95 °C for 20 s, annealing at 58 °C for 20 s, and extension at 72 °C for 1 min.

### 2.5. Cryosection and In Situ Hybridization

Spinyhead croaker gonads were fixed with 4% paraformaldehyde, and then were sliced into 6 μm. To investigate the subcellular location of *Cldnd* RNA, in situ hybridization (SISH) and dual fluorescence in situ hybridization (FISH), which could hybridize labeled probes with nucleic acids in cells or tissues, were performed as described [39,40]. Plasmid (pT-*Cldnd*) was constructed by inserting CDS of *Cldnd* into the pGEM-T vector. Then, pT-*Cldnd* was linearized for sense and antisense RNA probes from SP6 polymerase by using FITC RNA Labeling Kit (Roche, Basel, Switzerland). *Clvasa* probe labeled by digoxigenin (DIG) was prepared as described previously [37]. 

### 2.6. Preparation of Chimeric mRNAs and Microinjection

The recombinant plasmid of pCSpf*Cldnd* 3′ UTR (*gfp*-*Cldnd* 3′ UTR) was constructed by replacing the *Clvasa* 3′ UTR (amplified by primers *Clu-dnd* 3UF and 3UR) with *Cldnd* 3′ UTR in pCSpf*Clvasa* 3′ UTR (*gfp*-*Clvasa* 3′ UTR) [37]. The plasmid, pCSch*Drnos1* 3′ UTR (*rfp*-*Drnos1* 3′ UTR), was described [12]. Capped mRNAs used for microinjection were synthesized by using the mMessage Machine kit (Amibtion) from linearized *gfp*-*Cldnd* 3′ UTR and *rfp*-*Drnos1* 3′ UTR. Two chimeric mRNAs were co-injected at 100 ng/μL concentration into one-cell stage embryos from medaka for PGCs visualization.

### 2.7. Microscopy

Observation and photography were carried out on Nikon SMZ25 and Ni-E microscope with Nikon Ds-Ri2 camera (Nikon, Tokyo, Japan) as described [39].

## 3. Results

### 3.1. Cloning and Characterization of Cldnd

By the approach combining degenerate PCR with RACEs, a 1377 bp *Cldnd* complementary (c) DNA (GenBank accession No. MK547285) in full-length containing a 32 bp 5′ UTR, a 244 bp 3′ UTR and a 1098 bp CDS that encodes 366 amino acids was obtained (Figure S1). Multiple alignment analysis showed that ClDnd had a high identity with Dnd orthologs across species, ranging from 97.2% in large yellow croaker to 79.6% in medaka (Figure 1A). In addition, by the blast to the Conserved Domain Database (CDD) (https://www.ncbi.nlm.nih.gov/Structure/cdd/cdd.shtml), it was found that the deduced ClDnd protein carried six conserved domains or motifs, including N-terminal region (NR), RNA recognition motif (RRM) and four C-terminal regions (CR1-4), which were typically present in Dnd protein (Figure 1A and Figure S1).

The molecular phylogenetic tree demonstrated that Dnd proteins formed two distinct clades, which were the fish clade and the other vertebrates’, and ClDnd was the closest with that of the large yellow croaker in the fish sub-clade (Figure 1B). 

### 3.2. Cldnd RNA Is Specifically Expressed in Germ Cells

**Figure 2:**
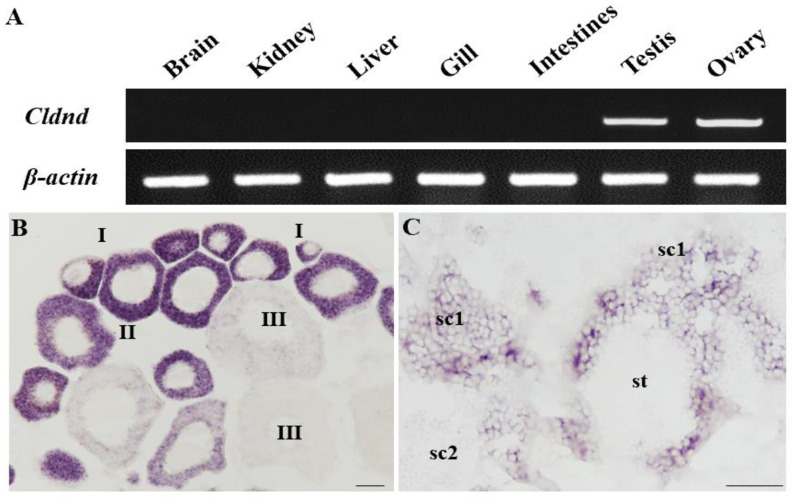
*Cldnd* RNA expression. (**A**) RT-PCR analysis of *Cldnd* in adult tissues. (**B**,**C**) Ovarian and testicular cryosections using antisense *Cldnd* probe and the signals were visualized by chromogenic staining. sc1 and sc2, primary and secondary spermatocytes; st, spermatid; I–III, stages of oocytes. Scale bar, 200 μm.

**Figure 3:**
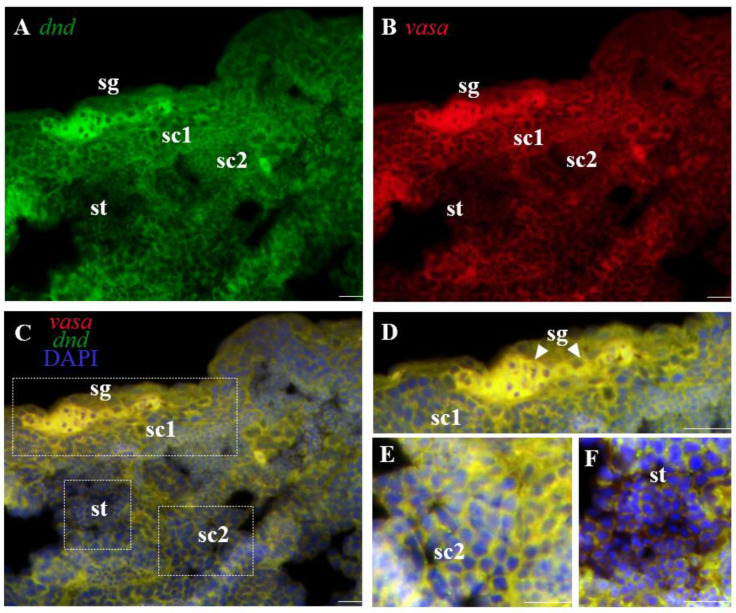
Co-localization of *Cldnd* and *Clvasa* mRNA in testis. Dual color FISH with *Cldnd* and *Clvasa* antisense RNA probes on testis and the signals were visualized by fluorescence staining. Nuclei were stained with DAPI (blue). (**A**) The signals were stained for the *dnd* RNA (green) by FISH. (**B**) The *vasa* signals were stained red. (**C**) The merges of *vasa* with *dnd* and DAPI. (**D**–**F**) Larger Magnification of panels C (white frame), highlighting the different cells. sg, spermatogonia; sc1 and sc2, primary and secondary spermatocytes; st, spermatid. Scale bar, 200 μm.

As described above, *dnd* is a gene which is specifically expressed in germ cells [41]. The RT-PCR analysis of seven distinct organs in *C. lucidus* revealed that only in the testis and ovary, bands corresponding to *Cldnd* RNA were shown, but absent in other somatic tissues such as the brain, kidney, liver, gill, and intestines (Figure 2A). Furthermore, SISH was carried out to investigate the subcellular localization of *Cldnd* RNA in the gonads. In the ovary, *Cldnd* signals were intensely expressed in oocyte I and were easily detected in oocyte II. As oogenesis progressed, the signals in stage III oocytes weakened and eventually vanished (Figure 2B). In the testis, *Cldnd* RNA was abundant in spermatogonia, then reduced markedly in primary spermatocytes and was faint in secondary spermatocytes, and finally disappeared in spermatozoon and sperm (Figure 2C and Figure 3A). On the contrary, the sense probe was unable to detect any signal (data not shown). According to the findings, *Cldnd* RNA was only found in male and female germ cells.

**Figure 4:**
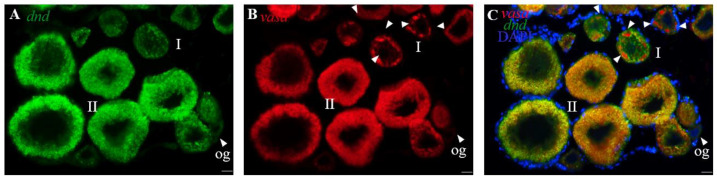
Co-localization of *Cldnd* and *Clvasa* mRNA in ovary. Dual color FISH with *Cldnd* and *Clvasa* antisense RNA probes on ovary and the signals were visualized by fluorescence staining. Nuclei were stained with DAPI (blue). (**A**) The signals were stained for the *dnd* RNA (green) by FISH. (**B**) The *vasa* signals were stained red. (**C**) The merges of *vasa* with *dnd* and DAPI. og, oogonia; I–II, stages of oocytes. Scale bar, 200 μm.

In several species, including *C. lucidus*, *vasa* is referred to as the best-characterized germ cell marker [37,42]. In this study, *Clvasa* was selected to explore the *Cldnd* RNA expression patterns. By FISH, *Cldnd* and *Clvasa* RNA showed the same expression pattern in the testis (Figure 3A–F). In the ovary, *Cldnd* RNA was as weak as *Clvasa* in oogonia, but it was uniformly distributed in the cytoplasm of oocyte I, while *Clvasa* aggregated into some particles (Figure 4A–C). 

### 3.3. Cldnd 3′ UTR Enables GFP to Express in the PGCs of Medaka Stably

**Figure 5:**
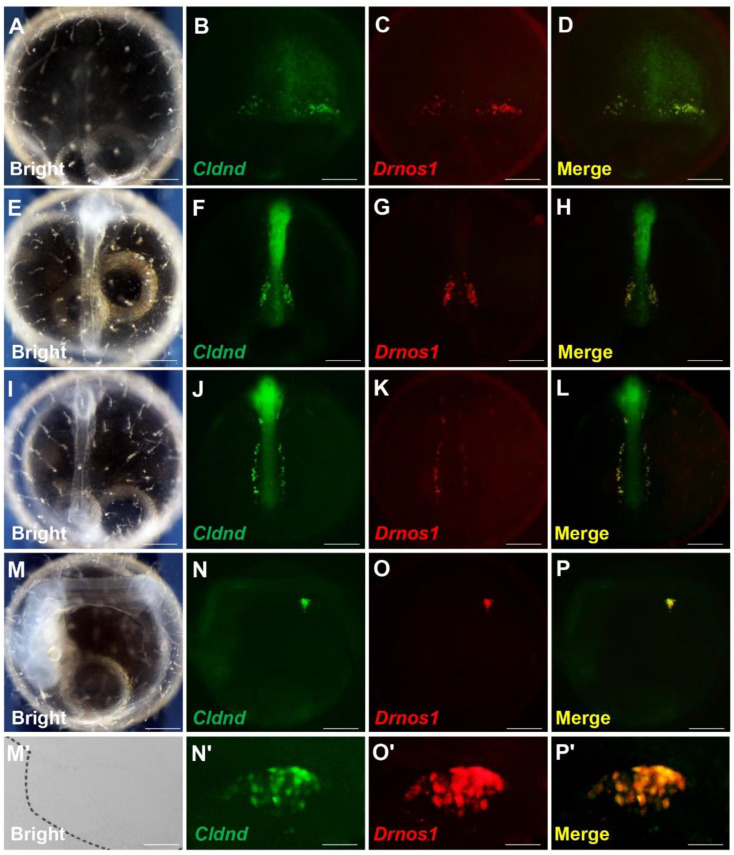
Visualization of PGCs by *Cldnd* 3′ UTR. (**A**–**P**) Medaka PGCs were visualized by co-injection of *gfp-Cldnd* 3′ UTR mRNA and *rfp-Drnos* 3′ UTR mRNA during embryogenesis. The merged images are shown on the right (**D**,**H**,**L**,**P**). (**M’**–**P’**) An isolated gonad was squashed and visualized at high magnification. Concentrations of injected mRNA are all 100 ng/μL. Scale bar, 200 μm.

There is a potential mechanism that enables foreign proteins to be stably and specifically expressed in germ cells under the protection of the 3′ UTR that is isolated from some germplasm-specific genes, of which the 3′ UTR of *nanos1* from zebrafish is the first candidate in fish, and has been widely verified and used for PGCs visualization in diverse species [43]. In order to confirm whether *Cldnd* 3′ UTR was responsible for the localization of mRNA and labeled PGCs, *gfp-Cldnd* 3′ UTR mRNA was injected into fertilized medaka embryos with *rfp-Drnos1* 3′ UTR mRNA which could effectively visualize PGCs [43]. At the early stage of embryogenesis, the GFP signals were distributed all over the cells (data not shown). Until the mid-gastrula stage, it turned into some intense particles and accumulated on the peripheral margin on both sides of the embryonic shield (body) (Figure 5B). At the somitogenesis stage, cells with positive GFP signal migrated dorsally aligned bilaterally along the trunk at the two sides of the embryonic body (Figure 5F,J). Finally, these cells migrated along the embryo body to the genital ridge, the position where gonads formed under the interaction of germ cells and mesoderm (Figure 5N). Meanwhile, the RFP signal (Figure 5C,G,K,O) displayed a similar expression pattern as GFP, suggesting that *gfp-Cldnd* 3′ UTR mRNA could label the PGCs of medaka (Figure 5D,H,L,P). When observed at high magnification, most of the PGCs with GFP signal was positive for RFP expression as well (Figure 5M′–P′).

## 4. Discussion

In this study, we obtain full-length of *Cldnd* cDNA and analyze its RNA expression in adult gonads. In addition, SISH and dual-color FISH demonstrate that *Cldnd* RNA is exclusively expressed in the germ cells of both sexes and shows a similar expression pattern with *Clvasa*. Surprisingly, medaka PGCs can be visualized by the injection of *gfp-Cldnd* 3′ UTR mRNA and *dnd* is identified as the second germline marker gene in *C. lucidus*. 

Dnd protein multiple alignment analysis manifests that ClDnd protein shows high identity to others, especially large yellow croaker. Moreover, ClDnd protein possesses the conserved motifs and domains (NR, RRM and CR1-4) similar to many examined fishes. RRM has been reported to be able to determine the subcellular localization of Dnd [44]. In zebrafish, the active site of Dnd protein exists in the C terminal domain which is required for the survival of PGCs [45]. Based on phylogenetic tree analysis of the Dnd protein, two main clades are found in vertebrates, and *C. lucidus* Dnd homologue is the closest to that of large yellow croaker in the fish sub-clade. 

In most vertebrates, *dnd* homologues are expressed only in both ovary and testis, except frog *dnd*, which is specific to the ovary [6]. Notably, in some teleost fish, the expression levels of *dnd* are higher in the ovary than testis [46,47,48]. However, in adults, the results of RT-PCR demonstrate that *Cldnd* RNA is exclusive and displays a similar expression level in both sexual gonads, which is consistent with the expression pattern in medaka [8] and zebrafish [4]. 

*Dnd* RNA as a marker for germ cells of both sexes has been identified in many teleost fish. Based on our previous reports in *C. lucidus*, we have identified germ cells marker gene *vasa* which is significant for germ cell development during gamogenesis. In the present study, SISH was performed to reveal the subcellular localization of *Cldnd* RNA, whose phenomenon is in keeping with zebrafish [4], medaka [8], turbot [13] and carp [21,22], suggesting that the *Cldnd* RNA expression is germ cell-specific. Moreover, dual color FISH is used to compare precisely the RNA expression patterns between *Cldnd* and *Clvasa*. The results of co-localization show that there is almost no difference between *Cldnd* and *Clvasa* in the testis, which is in line with the expression pattern in summer flounder (*Paralichthys dentatus*) [49]. In the ovary, *Cldnd* RNA is always uniformly distributed in the cytoplasm of germ cells, which differs from the specific expression of *Clvasa* in oocytes of stage I. These results indicate that *Cldnd* plays an important role in germ cell development.

Numerous studies have reported that the fusion of the *gfp/rfp* reporter gene and 3′ UTR of several germline-specific genes is very stable in PGCs but gradually degradative in somatic cells [50]. What more convincing explanation made for this biological characteristic is that the Dnd protein interacts with microRNA (miR-430) to protect gonadal-specific genes from degradation [1,13,51]. For example, *hub* 3′ UTR contains four noncanonical binding sites of miR-430 which is also a target of global miRNA-mediate repression. Interestingly, the function of germ-plasm specific genes is conserved widely across fish species. For instance, 3′ UTR of zebrafish *nanos1* can not only visualize its own PGCs but most of the examined fish, such as medaka, Chinese sturgeon, and salmon [1,9,39,52]. In this study, *gfp*-*Cldnd* 3′ UTR mRNA and *rfp*-*Drnos1* 3′ UTR mRNA are co-injected into medaka embryos and show a similar expression pattern in the PGCs during embryogenesis, which means that *gfp*-*Cldnd* 3′ UTR mRNA successfully marks medaka PGCs. *Cldnd* 3′ UTR is found in existence of several noncanonical miR-430 binding sites (-UCGUGAAA-) which may be responsible for the characteristic that eliminates the *gfp-Cldnd* 3′ UTR mRNA from somatic cells. Thus, *Cldnd* 3′ UTR is conserved for PGCs specific visualization.

In summary, these results manifest that *dnd* is an evolutionarily conserved germ cells marker and plays a vital role in the reproductive development of *C. lucidus*. The sequence structure, expression pattern, and function of 3′ UTR of *Cldnd* are highly similar to that in the examined species. These findings not only provide another germ cell maker but also benefit the functional and practical research on *Cldnd* and conservation of the *C. lucidus* population. 

## Figures and Tables

**Table 1:** Sequences of primers used in the present study.

Primer	Sequence (5′ to 3′ Direction)	Purpose
*Clu*-*dnd* DF	TGGGAGTTCAGGCTCATGATG	Degenerate primer
*Clu*-*dnd* DR	TCNACNARNACYTTYTTNGCCAT	
*Clu*-*dnd* 3NF	ACGCTGGAGGAAGCTTGGCAGGC	3′ RACE
*Clu*-*dnd* 3F	GAGATTCACTACAGCCACACCGAG	
*Clu*-*dnd* 5NR	CTTGAGTCAGCTTTGTATTGG	5′ RACE
*Clu*-*dnd* 5R	TGGTCCTCCATACTTCCTCTG	
*Clu*-*dnd* F	ATGATGGACCCCAAGCAGAGCC	RT-PCR
*Clu*-*dnd* R	GTTGGTGAACCGACTGTT	
*β*-*actin* F	TTTCAACAGCCCTGCCAT GTAC	Internal control
*β*-*actin* R	CCTCCAATCCAGACAGAGTATT	
*Clu*-*dnd* 3UF	ctcgagCTTGGGTTCAGAGGATATG	3′ UTR
*Clu*-*dnd* 3UR	ggtaccCCTTTTAAATCTCATTTA	

## Data Availability

Not applicable.

## Supplementary Materials

The following supporting information can be downloaded at: https://www.mdpi.com/article/10.3390/life12081226/s1, Figure S1: Nucleotide and deduced amino acid sequence of *Cldnd*; Figure S2: *gfp-dnd* 3′ UTR reporter.
