# Supplementary material for: Spinyhead Croaker Germ Cells Gene dnd Visualizes Primordial Germ Cells in Medaka
Source: Life (Basel). 2022 Aug 12;12(8):1226. doi: 10.3390/life12081226 (PMC9409898; doi:10.3390/life12081226)
Supplement: Supplementary file 1 [file life-1828734-supplementary.pdf › life-1828734-supplementary.pdf]

```
1     ACGCGGGGGCCGCGAAGGTAACAACGGTGGAGATGATGGACCCCAAGCAGAGCCAGGTACTCATCCTTAGGCGGATGCAGAAGCTGGAGACCTGGCTGAAAACAACCAATACAAAGCTGA
1                                     M  M  D  P  K  Q  S  Q  V  L  I  L  R  R  M  Q  K  L  E  T  W  L  K  T  T  N  T  K  L
121   CTCAAGTTAACGGCCAGAGGAAGTATGGAGGACCACCTAAAGTGTGGATGGGGTCCATTCCAGGAAACAACTGTGAGGTCTTCATCTCCCAAATCCCACTGGATGCCTACGAGGATCTGT
                     NR                                                                                      RRM
40    T  Q  V  N  G  Q  R  K  Y  G  G  P  P  K  V  W  M  G  S  I  P  G  N  N  C  E  V  F  I  S  Q  I  P  L  D  A  Y  E  D  L
241   TGATCCCCCTGTTCAGCTTGGCGGGGCCACTCTGGGAGTTCAGGCTCATGATGAATTTCAGTGGCCAGAACCGCGGTTTCGCCTATGCCAAATACGGATCTCCAGCCATAGCTAACGCCG
80    L  I  P  L  F  S  L  A  G  P  L  W  E  F  R  L  M  M  N  F  S  G  Q  N  R  G  F  A  Y  A  K  Y  G  S  P  A  I  A  N  A
361   CCATCCGCCTGCTGCATGGTCACATGGTGGAGCCTGGCTGTTACCTCGTCGTCCGCCACAGCATCGAGAAGAAACAGCTCTGTATCAAAGACCTGCCTGGCAAAACCAAGCAAGAGGACC
                                                                                                  CR1
120   A  I  R  L  L  H  G  H  M  V  E  P  G  C  Y  L  V  V  R  H  S  I  E  K  K  Q  L  C  I  K  D  L  P  G  K  T  K  Q  E  D
481   TGCTGAAGGTGCTGCGGGCACTGACGCATGGGGTGGAGCGAGTGTCTCTGAGACGTGAGTCCGGTATACCGGGGGTGTCTGCTGTAGTGGTCTTCTTAACCCACTACTCTGCTTCTATGG
160   L  L  K  V  L  R  A  L  T  H  G  V  E  R  V  S  L  R  R  E  S  G  I  P  G  V  S  A  V  V  V  F  L  T  H  Y  S  A  S  M
601   CCAAGAAGGTGCTGGTGGAAGCATTCAAGAAGCAGTTTGACTTGGACATCATCGTTCAGTGGGAGTCAACAATAAAGCCGAACCCTGACAAACCACATCGCACCGTCAACTTTCCACAGC
                         CR2
200   A  K  K  V  L  V  E  A  F  K  K  Q  F  D  L  D  I  I  V  Q  W  E  S  T  I  K  P  N  P  D  K  P  H  R  T  V  N  F  P  Q
721   CTTGTGTCCAGCGTCCTCGTGTGGCCTGCCCTCCACCCCCCGGTTTCTGCAGAGCAGTGGGAGGACCCACTTCCCCCAAGCACCCTCTCCCTTCACGCTCCATGAATTCCTCTTCCCGCC
240   P  C  V  Q  R  P  R  V  A  C  P  P  P  P  G  F  C  R  A  V  G  G  P  T  S  P  K  H  P  L  P  S  R  S  M  N  S  S  S  R
841   AGAGGCACCCAGTGGCCACAGCATGTTCAGCGTCCCCTGTGACGCTCCTGCAAAAGATGTGCAAGGCGGCCGGGGTTGGCGAGCCCGTCTATGAGATTCACTACAGCCACACCGAGTCTA
280   Q  R  H  P  V  A  T  A  C  S  A  S  P  V  T  L  L  Q  K  M  C  K  A  A  G  V  G  E  P  V  Y  E  I  H  Y  S  H  T  E  S
961   ATGGATTCCTCTATTTTACCTACCAGGTGTGTATCTCAGGGATAAGCCATGCCTTCAAAGGGGGCGTTATGACCTTGCCAGGACCTACTGCAGCCGCCACGCTGGAGGAAGCTTGGCAGG
                           CR3                                                                                CR4
320   N  G  F  L  Y  F  T  Y  Q  V  C  I  S  G  I  S  H  A  F  K  G  G  V  M  T  L  P  G  P  T  A  A  A  T  L  E  E  A  W  Q
1081  CTTCAGCCCAGCAGGTCGTGCTGAGGGTGAGGAACAGTCGGTTCACCAACTGACTTGGGTTCAGAGGATATGCATTTCACCTGCTTTTACAGCACTGTTCCAGGATCTGTTTATTTTGTT
360   A  S  A  Q  Q  V  V  L  R  V  R  N  S  R  F  T  N  *
1201  CATGATATAAAAAACGTTTGGTTTTATGTGGTTTGTTTTTTGCATGCTTTTATTAGTACTAGATTGGTTTGTGTTGTTTGTTCAGATTGTTTTTATTGTGTGTTACTGTTGGATCGCTTC
1321  CTCATTTTAATTAAATGAGATTT
```

Figure S1. Nucleotide and deduced amino acid sequence of *Cldnd*. Start and stop codon signal were shown in bold. Underlined parts were amino acid sequences used for degenerate primers, specific sequences for PCR amplification of the full-length cDNA (arrows depicting directions) and the coding sequence that was cloned in pGEMT-Easy for RNA probe preparation.

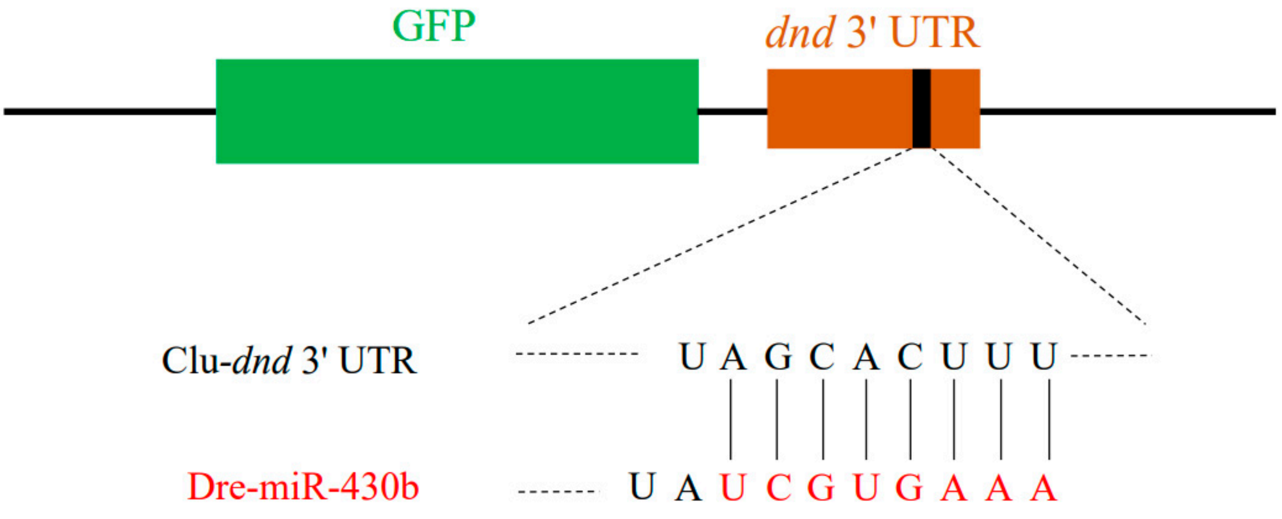

Figure S2. *gfp-dnd* 3' UTR reporter. Putative base pairing of Dre-miR-430b with the *C. lucidus dnd* 3' UTR. The conservative sequence of miR-430 is red.
